# Problematic social media use mediates the effect of cyberbullying victimisation on psychosomatic complaints in adolescents

**DOI:** 10.1038/s41598-024-59509-2

**Published:** 2024-04-29

**Authors:** Prince Peprah, Michael Safo Oduro, Godfred Atta-Osei, Isaac Yeboah Addo, Anthony Kwame Morgan, Razak M. Gyasi

**Affiliations:** 1 Social Policy Research Centre, University of New South Wales, Sydney, Australia; 2 Centre for Primary Health Care and Equity, University of New South Wales, Sydney, Australia; 3 Pfizer Research and Development, PSSM Data Sciences, Pfizer, Inc., Connecticut, USA; 4 Centre for Disability and Rehabilitation Studies, Kwame Nkrumah University of Science and Technology, Kumasi, Ghana; 5 Centre for Social Research in Health, University of New South Wales, Sydney, Australia; 6 Concord Clinical School, University of Sydney, Sydney, Australia; 7 Department of Planning, Kwame Nkrumah University of Science and Technology, Kumasi, Ghana; 8 African Population and Health Research Center, Nairobi, Kenya; 9 National Centre for Naturopathic Medicine, Faculty of Health, Southern Cross University, Lismore, NSW Australia

**Keywords:** Adolescent well-being, Psychosomatic complaints, Public health, Cyberbullying victimisation, Sleep quality, Social media use, Public health, Risk factors

## Abstract

Adolescent psychosomatic complaints remain a public health issue globally. Studies suggest that cyberbullying victimisation, particularly on social media, could heighten the risk of psychosomatic complaints. However, the mechanisms underlying the associations between cyberbullying victimisation and psychosomatic complaints remain unclear. This cross-cultural study examines the mediating effect of problematic social media use (PSMU) on the association between cyberbullying victimisation and psychosomatic complaints among adolescents in high income countries. We analysed data on adolescents aged 11–16.5 years (weighted N = 142,298) in 35 countries participating in the 2018 Health Behaviour in School-aged Children (HBSC) study. Path analysis using bootstrapping technique tested the hypothesised mediating role of PSMU. Results from the sequential binary mixed effects logit models showed that adolescents who were victims of cyberbullying were 2.39 times significantly more likely to report psychosomatic complaints than those who never experienced cyberbullying (AOR = 2.39; 95%CI = 2.29, 2.49). PSMU partially mediated the association between cyberbullying victimisation and psychosomatic complaints accounting for 12% ($\beta$ = 0.01162, 95%CI = 0.0110, 0.0120) of the total effect. Additional analysis revealed a moderation effect of PSMU on the association between cyberbullying victimisation and psychosomatic complaints. Our findings suggest that while cyberbullying victimisation substantially influences psychosomatic complaints, the association is partially explained by PSMU. Policy and public health interventions for cyberbullying-related psychosomatic complaints in adolescents should target safe social media use.

## Introduction

Adolescence is noted to be a critical developmental stage, with many problems, including loneliness^1^, poor friendships, an adverse class climate, school pressure^2^, suicidal ideation and attempts, and psychosomatic complaints^3^. Psychosomatic complaint is a combination of physical ailments (i.e., headaches, stomach aches, fatigue, and muscle pain) caused or exacerbated by psychological factors such as stress, irritability, anxiety, or emotional distress^4,5^. Psychosomatic complaints are common among adolescents, and recent estimates indicate that the global prevalence of psychosomatic complaints ranges between 10 and 50%^6^. Also, an increase in self-reported psychosomatic complaints and related mental health complaints have been reported in adolescents from high-income countries^7,8^. The high prevalence of psychosomatic complaints is of concern as psychosomatic complaints have severe implications for multiple detrimental health outcomes, healthcare expenditure, and quality of life of young people^9^. Thus, it is of utmost importance to identify the proximate risk factors for psychosomatic complaints among young people to aid in developing targeted interventions to reduce the incidence of psychosomatic complaints, mainly in high-income countries.

While extant research has identified risk factors for psychosomatic complaints, including malnutrition, low physical activity, and poor parental guidance^10–12^, one understudied but potentially important risk factor is cyberbullying victimisation. Cyberbullying victimisation is an internet-based aggressive and intentional act of continually threatening, harassing, or embarrassing individuals who cannot defend themselves using electronic contact forms such as emails, text messages, images, and videos^13,14^. Indeed, being typical of interpersonal interactions, cyberbullying victimisation has shown a rising trend, particularly during adolescence^15^. International literature has shown the prevalence of cyberbullying victimisation to be between 12 and 72% among young people^14,16^. It may be hypothesised that cyberbullying victimisation potentially increases the risk of psychosomatic complaints through factors such as problematic social media use (PSMU)^17,18^. However, studies are needed to identify whether and the extent to which such factors mediate the potential association of cyberbullying victimisation with psychosomatic complaints among young people.

Given this background, the present study aimed to investigate the association between cyberbullying victmisation and psychosomatic complaints in 142,298 young people aged 11–16.5 years from 35 high-income countries. A further aim was to quantify how PSMU mediates the association between cyberbullying victimisation and psychosomatic complaints.

### Cyberbullying victimisation and adolescents’ psychosomatic complaints

Research has consistently shown that cyberbullying victimisation significantly impacts adolescents’ mental health^19^. For example, Kowalski and Limber^20^ found that cyberbullying victimisation is associated with increased levels of depression, anxiety, and social anxiety, as well as psychosomatic complaints, such as fatigue and muscle tension. Further, studies have shown that cyberbullying victimisation and perpetration can lead to a variety of physical, social, and mental health issues, including substance abuse and suicidal thoughts and attempts^21–24^. Furthermore, cyberbullying victimisation is strongly associated with suicidal thoughts and attempts, regardless of demographic factors like gender or age^21,25^. These findings underscore the urgent need for interventions that address the mental health consequences of cyberbullying, particularly for adolescents, who are most vulnerable to its harmful effects. The findings also suggest that cyberbullying might be a potential underlying predictor of higher psychosomatic disorders among adolescents. This present study, therefore, hypothesises **that H1:** there is a statistically significant association between cyberbullying victimisation (X) and psychosomatic complaints (Y) (total effect).

### The role of adolescents’ PSMU

Problematic Social Media Use (PSMU), a subtype of problematic internet use, refers to the uncontrolled, compulsive or excessive engagement with social media platforms such as Facebook and Twitter, characterised by addictive behaviours like mood alteration, withdrawal symptoms, and interpersonal conflicts. This pattern of social media usage can result in functional impairments and adverse outcomes^26^. Scholars and professionals have shown great concern about the length of time adolescents spend on social media. Studies have observed that (early) adolescence could be a crucial and sensitive developmental stage in which adolescent users might be unable to avoid the harmful impacts of social media use^27^. According to current research, PSMU may increase adolescents’ exposure to cyberbullying victimisation, which can have severe consequences for their mental health^28–30^. Similarly, an association between PSMU and physical/somatic problems, as well as somatic disorders, has been established in many studies^31,32^. Hanprathet et al.^33^ demonstrated the negative impact of problematic Facebook use on general health, including somatic symptoms, anxiety, insomnia, depression, and social dysfunction. According to Cerutti et al.^34^, adolescents with problematic social media usage have more somatic symptoms, such as stomach pain, headaches, sore muscles, and poor energy, than their counterparts. Hence, inadequate sleep may be associated with PSMU, harming both perceived physical and mental health^35,36^. Again, supporting the above evidence, the relationship between PSMU, well-being, and psychological issues have been highlighted in meta-analytic research and systematic reviews^27,31,37,38^. Thus, this study proposes the following hypothesis: **H2:** there is a specific indirect effect of cyberbullying victimisation (X) on psychosomatic complaints (Y) through PSMU (M1) (indirect effect a_1_b_1_).

## Methods

### Study, sample, and procedures

This study used data from the 2018 Health Behaviour in School-aged Children (HBSC) survey conducted in 35 countries and regions across Europe and Canada during the 2017–2018 academic year^39^. The HBSC research team/network is an international alliance of researchers collaborating on a cross-national survey of school students. The HBSC collects data every four years on 11-, 13- and 15- year-old adolescent boys’ and girls’ health and well-being, social environments, and health behaviours. The sampling procedure for the 2018 survey followed international guidelines^40,41^. A systematic sampling method was used to identify schools in each region from the complete list of both public and private schools. Participants were recruited through a cluster sampling approach, using the school class as the primary sampling unit^42^. Some countries oversampled subpopulations (e.g., by geography and ethnicity), and standardised weights were created to ensure representativeness of the population of 11, 13, and 15 years^43^. Questionnaires were translated based on a standard procedure to allow comparability between the participating countries. Our analysis used data from 35 countries and regions with complete data on cyberbullying victimisation, PSMU, and psychosomatic complaints. The study complies with ethical standards in each country and follows ethical guidelines for research and data protection from the World Health Organisation and the Organisation for Economic Co-operation and Development. Depending on the country, active or passive consent was sought from parents or legal guardians and students which was checked by teachers to participate in the study. The survey was conducted anonymously and participation in the study was voluntary for schools and students. Schools, children and adolescents could refuse to participate or withdraw their consent until the day of the survey. Moreover, all participating students were free to cease filling out the questionnaire at any moment, or to answer only selected questions. More detailed information on the methodology of the HBSC study including ethics and data protection can be found elsewhere^44,45^.

## Measures

### Outcome variable: psychosomatic complaints

Psychosomatic complaints was assessed by one collective item asking students how often they had experienced the following complaints over the past six months: headache, stomach aches, feeling low, irritability or bad mood, feeling nervous, dizziness, abdominal pain, sleep difficulty, and backache. Response options included: about every day, more than once a week, about every week, about every month, and rarely or never. This scale has sufficient test–retest reliability and validity^46^, good internal consistency (Cronbach’s a = 0.82)^47^, and has been applied in several multiple country analyses^48,49^. The scale is predictive of emotional problems and suicidal ideation in adolescents^50,51^. For our analysis, the scale was dichotomised with two or more complaints several times a week or daily coded as having psychosomatic complaints^47,49^.

### Exposure variable: Cyberbullying victimisation

Cyberbullying victimisation is the exposure variable in this study. Thus, the exposure variable pertains to only being a victim of cyberbullying and does not include perpetration of cyberbullying. Students were first asked to read and understand a short definition of cyberbullying victimisation. They were then asked how often they were bullied over the past two months (e.g., someone sending mean instant messages, emails, or text messages about you; wall postings; creating a website making fun of you; posting unflattering or inappropriate pictures of you online without your permission or sharing them with others). Responses included: “*I have not* *been* cyberbullied”, “once or twice”, “two or three times a month”, “about once a week”, and “several times a week”. These were dichotomised into “never" or “once or more". This measure of bullying victimisation has been validated across multiple cultural settings^43,52–54^.

### Mediating variable

*Problematic social media use* (PSMU) was assessed with the Social Media Disorder Scale (Cronbach’s a = 0.89)^55^. The scale contains nine dichotomous (yes/no) items describing addiction-like symptoms, including preoccupation with social media, dissatisfaction about lack of time for social media, feeling bad when not using social media, trying but failing to spend less time using social media, neglecting other duties to use social media, frequent arguments over social media, lying to parents or friends about social media use, using social media to escape from negative feelings, and having a severe conflict with family over social media use. In this study, the endorsement of six or more items indicated PSMU as evidence suggests that a threshold of six or more is an indicative of PSMU^54,56^. This scale has been used across cultural contexts^43,52,54^.

### Covariates

Informed by previous studies^43,54,57^, the analysis controlled for theoretically relevant confounders, including sex (male/female) and age. Family affluence/socio-economic class was assessed using the Relative Family Affluence Scale, a validated six-item measure of material assets in the home, such as the number of vehicles, bedroom sharing, computer ownership, bathrooms at home, dishwashers at home, and family vacations)^56,58^. Finally, parental and peer support were measured using an eight item-measure^59^. Responses were recorded on a 7-point Likert scale (ranging from 0 indicating very strongly disagree to 6 indicating very strongly agree).

### Statistical analysis

Region-specific descriptive statistics were calculated to describe the sample. Next, Pearson’s Chi-squared association test with Yates’ continuity correction was performed to examine plausible associations between psychosomatic complaints and other categorical study variables. Also, to account for the regional clustering or unobserved heterogeneity observed in the analytic sample, sequential mixed effect binary logit models with the inclusion of a random intercept were fitted to further examine the associations between psychosomatic complaints and cyberbullying victimisation as well as other considered covariates. Furthermore, a parallel mediator model was fitted to evaluate the specified hypothesis and understand the potential mechanism linking cyberbullying victimisation and psychosomatic complaints. More specifically, cyberbullying victimisation (X) was modelled to directly influence psychosomatic complaints (Y) and indirectly via PSMU (M). Since core variables were binary, paths could be estimated with a sequence of three logit equations:^60,61^

1 $${\text{logit}}\left( {P\left( {Y = 1{|}x} \right)} \right) = i_{1} + cX + \varepsilon_{1} ;$$

2 $${\text{logit}}\left( {P\left( {M = 1{|}x} \right)} \right) = i_{2} + aX + \varepsilon_{2} ;$$

3 $${\text{logit}}\left( {P\left( {Y = \left. 1 \right|x} \right)} \right) = i_{3} + c^{\prime}X + bM + ~\varepsilon _{3} ;$$

where, ${i}_{1}$, ${i}_{2}$, and ${i}_{3}$ represent the intercept in the respective equations. The path coefficient, c, in Eq. (1) represents the total effect of predictor X on outcome *Y*. In Eq. (2), the path coefficient a denotes the effect of predictor *X* on the mediator *M*. Also, the *c'* parameter in Eq. (3) represents the direct effect of the predictor *X* on the response *Y*, adjusting for the mediator *M*. Lastly, the path coefficient *b* coefficient in Eq. (3) represents the indirect effect of the mediator *M* on the outcome *Y*, when adjusting for the predictor *X*. These logit models provide effect estimates on the log-odds scale, and thus can be transformed into odds ratios. Each model was adjusted for the potential confounding variables.

All statistical analyses were performed using R Software (v4.1.2; R Core Team 2021) with $\alpha$ = *0.05* as the significance level. More specifically, the package “mediation” in R^62^ was used for the mediation analysis to estimate direct, indirect, and total effects. Inference is based on a non-parametric, 95% bias-corrected and accelerated (BCa) bootstrapped confidence interval^63,64^. Bootstrapping for indirect effects was set at 1000 samples, and once the 95% bootstrapped CI of the mediation effects did not include zero (0), it was deemed statistically significant. We also conducted further analysis by including an interaction between cyberbullying victimisation and PSMU to obtain insights analogous to the mediation model.

### Ethics approval and consent to participate

The research was exclusively based on data sourced from the World Bank, which adheres to rigorous ethical standards in its data collection processes. Therefore, no separate ethical approval was sought or deemed necessary. Ethical approval was not required for this study since the data used for this study are secondary data. Necessary permissions and survey data were obtained from the World Bank. The World Bank data collection process upheld ethical standards and relevant guidelines in the research process including informed consent from all subjects and/or their legal guardian(s).

## Results

### Preliminary analyses

The final analytic sample comprised complete information on 142,298 adolescents from 35 high-income countries (Table 1). The median age of the sample was 13.6 years. Most participants resided in Wales (6.26%) and the Czech Republic (6.16%). Notably, the prevalence of cyberbullying victimisation was 26.2%, and the majority (53%) were females. As observed in Table 2, 84.6% of the participants self-reported high levels of psychosomatic complaints. Furthermore, among the participants who experienced PSMU, about 81.16% reported high levels of psychosomatic complaints. About 84.47% of the participants indicated receiving parental and peer support (see Table 2).

**Table 1:** Country/region-wise sample characteristics.

Per region sample characteristics		
Country/Region	Sample (n)	Sample Percentage (%)
Albania	1270	0.890
Austria	3249	2.280
Azerbaijan	2886	2.030
Belgium (Vlaamse Gewest)	3250	2.280
Belgium (Wallone)	3713	2.610
Canada	7804	5.480
Czech	8760	6.160
Germany	3269	2.300
Estonia	4095	2.880
Spain	3450	2.420
France	6236	4.380
England	2430	1.710
Scotland	3852	2.710
Wales	8902	6.260
Georgia	2962	2.080
Greece	3352	2.360
Croatia	3224	2.270
Hungary	3191	2.240
Ireland	2756	1.940
Israel	4633	3.260
Iceland	5625	3.950
Italy	3434	2.410
Kazakhstan	2867	2.010
Lithuania	3290	2.310
Luxembourg	2666	1.870
Moldova	3553	2.500
Malta	1941	1.360
Netherlands	4285	3.010
Portugal	5110	3.590
Romania	3571	2.510
Serbia	2861	2.010
Russian	2978	2.090
Sweden	3002	2.110
Slovenia	4357	3.060
Turkey	4280	3.010
Ukraine	5194	3.650
Total (N)	142,298	100

**Table 2:** Contingency table of psychosomatic complaints and core study variables and corresponding Chi-Square tests.

Variables	Levels/categories	Psychosomatic complaints (%, n)		Chi-square test
		Low	High	${\chi }^{2}$ Statistic, P-value
Sex	Male	19.340% ((12,961)	80.660% (54,065)	1471.6, *p* < 0.001
	Female	11.970% (9013)	88.030% (66,259)	
Cyberbullying victimisation	Never	17.850% ((18,746)	82.150% (86,268)	1780.2, *p* < 0.001
	Once or more	8.660% (3228)	91.340% (34,056)	
Parental and peer support	Low	14.000% (1125)	86.000% (6912)	13.494, *p* = 0.029
	High	15.530% (20,849)	84.470% (113,412)	
Family affluence	Lowest 20%	16.420% (4347)	83.580% (22,130)	23.738, *p* < 0.001
	Medium 60%	15.23% (13,512)	84.77% (75,226)	
	Highest 20%	15.190% (4115)	84.810% (22,968)	
PSMU	Yes	18.840% (18,714)	81.160% (80,595)	2912.7, *p* < 0.001
	No	7.580% (3260)	92.420% (39,729)	

Notes: PSMU, problematic social media use.

### Main analyses

**Figure 1:**
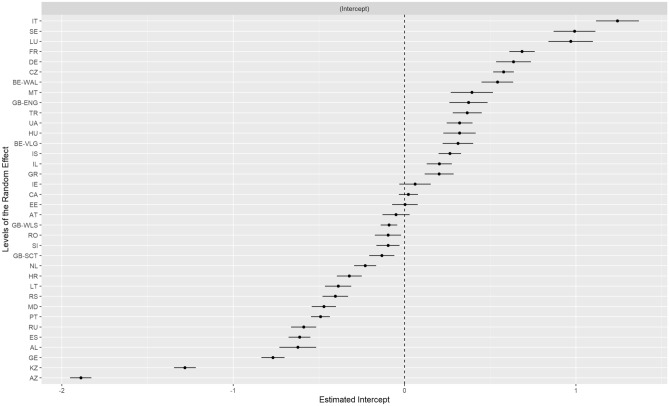
A caterpillar plot of empirical Bayes residuals of the models for the random intercept, region/country. This represents individual effects for each region/country. Region or country abbreviations in the figure are as follows: [AL] Albania, [AZ] Azerbaijan, [AT] Austria, [BE-VLG] Vlaamse Gewest (Belgium), [BE-WAL] Wallone, Région (Belgium), [CA] Canada, [CZ] Czech Republic, [DE] Germany, [EE] Estonia, [CA] Canada, [ES] Spain, [FR] France, [GB-ENG] England, [GB-SCT] Scotland, [GB-WLS] Wales, [GE] Georgia, [GR] Greece, [HR] Croatia, [HU] Hungary, [IE] Ireland, [IL] Israel, [IS] Iceland, [IT] Italy, [KZ] Kazakhstan, [LT] Lithuania, [LU] Luxembourg, [MD] Moldova, [MT] Malta, [NL] Netherlands, [PT] Portugal, [RO] Romania, [RS] Serbia, [RU] Russia, [SE] Sweden, [SI] Slovenia, [TR] Turkey, [LU] Luxembourg and [UA] Ukraine.

Results from the sequential binary mixed effects logit model are shown in Table 3. In the first step, we included only cyberbullying victimisation in the model. We found that cyberbullying victims were 2.430 times more likely to report psychosomatic complaints than those who were not cyberbullied (OR = 2.430; 95%CI = 2.330, 2.530). The second step included sex, PSMU, parental and peer support, and family affluence as covariates. We found that cyber bullying victims were 2.390 times significantly more likely to report psychosomatic complaints than those who never experienced cyberbullying (AOR = 2.390; 95%CI = 2.29, 2.49). Additionally, the third model, which is an additional analysis involved the inclusion of an interaction between and cyberbullying victimisation and PSMU. The results showed that PSMU moderates the association between cyberbullying victimisation and psychosomatic complaints. Adolescents who were cyberbullied but did not report PSMU had reduced odds of psychosomatic complaints compared to those with PSMU (AOR = 1.220; 95%CI = 1.110–1.350). Furthermore, a caterpillar plot of empirical Bayes residuals of the models for the random intercept, region/country is obtained and shown in Fig. 1. This represents individual effects for each country and offers additional insights into the extent of psychosomatic complaints heterogeneity across different countries. The plots visually demonstrates that regional variation for psychosomatic complaints does exist.

**Table 3:** Binary mixed effects binary logit modelling results.

Model 1: Simple binary mixed effects logistic regression results with bullying victimisation as sole predictor			
Fixed effects results			
Variable	OR	OR 95% CI	P-value
(Intercept)	4.800	3.920–5.880	< 0.001
Cyberbullying victimisation (Ref = Never)			
Once or More	2.430	2.330–2.530	< 0.001
Random effects			
Within region variance	3.290		
Between region variance	0.390		
Intra class correlation	0.100		
No of clusters	36.00		
Random intercept Std. Dev.	0.621		
Nakagawa & Schielzeth Conditional ${R}^{2}$	0.140		

Model 2: Mixed effects multiple binary logistic regression results with bullying victimisation and other considered covariates			
Fixed effects results			
Variable	AOR	OR 95% CI	P-value
(Intercept)	0.410	0.320–0.530	< 0.001
Cyberbullying victimisation (Ref = Never)			
Once or more	2.390	2.290–2.490	< 0.001
Age	1.230	1.220–1.240	< 0.001
Sex (Ref = Male)			
Female	1.840	1.790–1.900	< 0.001
Parental and Peer Support (Ref: Low)			
High	0.980	0.910–1.050	0.532
Family affluence (Ref: Lowest 20 pct)			
Medium 60 pct	1.100	1.060–1.140	< 0.001
Highest 20 pct	1.140	1.080–1.200	< 0.001
PMSU (Ref: Yes)			
No	0.400	0.380–0.410	< 0.001
Random effects			
Within region variance	3.290		
Between region variance	0.410		
Intra class correlation	0.110		
No of clusters	36.000		
Random intercept Std. Dev.	0.643		
Nakagawa & Schielzeth Conditional ${R}^{2}$	0.140		

Model 3: Mixed effects multiple binary logistic regression results with cyberbullying victimisation and PMSU interaction adjusted			
Fixed effects results			
Variable	AOR	OR 95% CI	P-value
(Intercept)	0.430	0.330–0.560	< 0.001
Cyberbullying victimisation (Ref = Never)			
Once or More	2.050	1.880–2.230	< 0.001
Age	1.230	1.220–1.240	< 0.001
Sex (Ref = Male)			
Female	1.840	1.790–1.900	< 0.001
Parental and Peer Support (Ref: Low)			
High	0.980	0.91–1.050	0.52
Family Affluence (Ref: Lowest 20 pct)			
Medium 60 pct	1.100	1.060–1.150	< 0.001
Highest 20 pct	1.140	1.080–1.200	< 0.001
PMSU (Ref: Yes)			
No	0.380	0.360–0.400	< 0.001
BV (Ref: Never) *PMSU (Ref: Yes)			
BV (Yes)*PMSU(No)	1.220	1.110–1.350	< 0.001
Random effects			
Within region variance	3.290		
Between region variance	0.410		
Intra class correlation	0.110		
No of clusters	36.000		
Random tntercept Std. Dev.	0.643		
Nakagawa & Schielzeth Conditional ${R}^{2}$	0.233		

Note: PSMU, problematic social media use.

**Figure 2:**
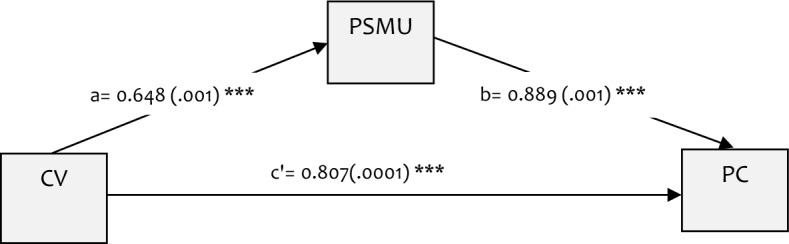
A parallel mediation model of the influence of PSMU on the association between Cyberbullying Victimisation and Psychosomatic Complaints. a = path coefficient of the effect of exposure on the mediator. b = path coefficient of the effect of the mediator on the outcome. c’ = path coefficient of the direct effect of the exposure on outcome. CV, cyberbullying victimisation. PC, psychosomatic complaints.

Figure 2 shows the adjusted parallel mediation results. The effect of cyberbullying victimisation on psychosomatic complaints was significantly mediated by PSMU. The paths from cyberbullying victimisation to PSMU (a: $\beta$=0.648, p < 0.001), PSMU to psychosomatic complaints (b: $\beta$=0.889, p < 0.001), and that of cyberbullying victimisation to 0.8069 (c′: $\beta$=0.051, p < 0.001) were also statistically significant.

### Bootstrapping test of mediating effects

The total, direct, and indirect effects of the mediation model based on nonparametric bootstrap are presented in Table 4. We observe that the estimated CI did not include zero (0) for any effects. This observation suggests a statistically significant indirect effect of cyberbullying victimisation on psychosomatic complaints via PSMU ($\beta$ = 0.01162, 95%CI = 0.0110, 0.0120), yielding 12% of the total effect.

**Table 4:** Bootstrapping test of mediating effects.

Path Model	Effect	Bootstrap Estimate	Bootstrap CI	P-value	Percentage Mediated
Cyberbullying victimisation → Problematic social media use → Psychosomatic complaints	Total	0.097	(0.093,0.099)	*p* < *0.001*	12%
	Direct	0.085	(0.082, 0.087)	*p* < *0.001*	
	Indirect	0.012	(0.011, 0.012)	*p* < *0.001*	

## Discussion

### Key findings

This cross-cultural study examined the direct and indirect associations of cyberbullying victimisation with psychosomatic complaints via PSMU among adolescents. The results showed that cyberbullying victimisation independently influenced the experience of psychosomatic complaints. Specifically, adolescents who were victims of cyberbullying were more than two times more likely to report psychosomatic complaints. Crucially, our mediation analyses indicated that PSMU explain approximately 12% of the association between cyberbullying victimisation and psychosomatic complaints. In a further analysis, PSMU moderated the association between cyberbullying victimisation and psychosomatic complaints. This study is the first to examine the direct and indirect associations between cyberbullying victimisation and psychosomatic complaints through PSMU in adolescents across multiple high-income countries.

### Interpretation of the findings

Our results confirmed the first hypothesis that there is a statistically significant direct association between cyberbullying victimisation and psychosomatic complaints. Thus, we found that cyberbullying independently directly affected the adolescents' experience of psychosomatic complaints. Previous studies have mainly focused on the direct effect of traditional face-to-face bullying on psychosomatic complaints^20,65^ or compared the impact of traditional face-to-face bullying to cyberbullying concerning mental health^19,66–69^. A systematic review of traditional bullying and cyberbullying victimisation offers a comprehensive synthesis of the consequences of cyberbullying on adolescent health^19^. Another review suggested that cyberbullying threatened adolescents’ well-being and underscored many studies that have demonstrated effective relationships between adolescents’ involvement in cyberbullying and adverse health outcomes^70^. Other population-based cross-sectional studies have similarly shown that victims of cyberbullying experience significant psychological distress and feelings of isolation, which can further exacerbate their physical and mental health challenges^22,71,72^. The present study builds on the previously published literature by highlighting the effect of cyberbullying victimisation on adolescent psychosomatic complaints and the extent to which the association is mediated by PSMU.

Consistent with the second hypothesis, we found that PSMU mediated about 12% of the association between cyberbullying victimisation and psychosomatic complaints in this sample. While studies on the mediational role of PSMU in the relationship between cyberbullying victimisation and psychosomatic complaints are limited, evidence shows significant interplay among PSMU, cyberbullying victimisation, and psychosomatic complaints. For example, a study of over 58,000 young people in Italy found that PSMU was associated with increased levels of multiple somatic and psychological symptoms, such as anxiety and depression.^73^ Another study of 1707 adolescents in Sweden found that cyberbullying victimisation was associated with increased depressive symptoms and the lowest level of subjective well-being^74^.

Other possible mediators of the cyberbullying victimisation-psychosomatic complaints association may include low self-esteem, negative body image, emotion regulation difficulties, social support, and personality traits such as neuroticism and impulsivity^20,67,72,75,76^. For example, Schneider et al.^75^ have shown that emotional distress could increase psychosomatic symptoms such as headaches, stomach aches, and muscle tension. In addition, social isolation can lead to social withdrawal and a decreased sense of belonging^78,79^. Therefore, it is essential to explore these variables further and develop effective interventions and prevention strategies to address these interrelated factors and reduce their negative impact on adolescent health and well-being.

In a further analysis, the results show that PSMU does not only mediate but also moderate the association between cyberbullying victimisation and psychosomatic complaints among adolescents. Specifically, cyberbullied adolescents with no report of PSMU had reduced likelihoods of experiencing psychosomatic complaints compared to those with PSMU. This result is interesting and could be due to several factors. First, individuals with PSMU may already be experiencing heightened levels of psychological distress due to their excessive social media use, making them more vulnerable to the negative effects of cyberbullying^80–82^. For instance, excessive time spent on social media, particularly in activities such as comparing oneself to others or seeking validation through likes and comments, has been linked to increased psychological distress^83,84^. Conversely, the finding that cyberbullied adolescents without PSMU had reduced likelihoods of experiencing psychosomatic complaints compared to those with PSMU suggests a protective effect of lower social media use. Adolescents who are not excessively engaged with social media may have fewer opportunities for exposure to cyberbullying and may also have healthier coping strategies in place to deal with any instances of online victimisation^43,85,86^.

The results suggest that professionals in the fields of education, counselling, and healthcare should prioritise addressing the issue of cyberbullying victimisation when assessing the physical and psychological health of adolescents. Evidently, adolescents who experience cyberbullying require support. Thus, proactive measures are essential, and support could be provided by multiple professional communities that serve adolescents and young people in society, such as educational, behavioural health, and medical professionals. Sensitive inquiry regarding cyberbullying experiences is necessary when addressing adolescent health issues such as depression, substance use, suicidal ideation, and somatic concerns^19^. Our findings underscore the need for comprehensive, school-based programs focused on cyberbullying victimisation prevention and intervention.

## Strengths and limitations

The study's main strength lies in the use of a large sample size representing multiple countries in high income countries. This large sample size improved the representativeness and veracity of our findings. The complex research approach helps advance our understanding of the interrelationships between cyberbullying victimisation, PSMU, and psychosomatic complaints among adolescents. However, the study has its limitations. First, the cross-sectional design does not allow directionality and causal inferences. Second, retrospective self-reporting for the critical study variables could lead to recall and social desirability biases. Third, the presence of residual and unobserved confounders, despite adjusting for some covariates, can be considered a limitation of this study. Further research is needed to confirm these findings and better understand how PSMU mediates the relationship between cyberbullying victimisation and psychosomatic complaints.

## Conclusions

This study has provided essential insights into the interrelationships between cyberbullying victimisation, PSMU, and psychosomatic complaints among adolescents in high income countries. The findings suggest that cyberbullying is directly associated with psychosomatic complaints and that PSMU significantly and partially mediates this association. This study also highlights the importance of addressing cyberbullying victimisation and its negative impact on adolescent health and emphasises the need to address PSMU. Overall, the study underscores the importance of promoting healthy online behaviour and providing appropriate support for adolescents who experience cyberbullying victimisation. Further studies will benefit from longitudinal data to confirm our findings.

## Data Availability

The data that support the findings of this study are available from the World Bank, but restrictions apply to the availability of these data, which were used under license for the current study and so are not publicly available. Data are, however, available from the corresponding author (anthoniomorgano280@gmail.com) upon reasonable request and with permission of the World Bank.
